# The effects of different types of aquatic exercise training interventions on a high-fructose diet-fed mice

**DOI:** 10.7150/ijms.52347

**Published:** 2021-01-01

**Authors:** Yi-Ju Hsu, Mon-Chien Lee, Chi-Chang Huang, Chun-Sheng Ho

**Affiliations:** 1 Graduate Institute of Sports Science, National Taiwan Sport University, Taoyuan 333325, Taiwan; 2 Department of Physical Therapy, College of Medical and Health Science, Asia University, Taichung 41354, Taiwan; 3 Division of Physical Medicine and Rehabilitation, Lo-Hsu Medical Foundation, Inc., Lotung Poh-Ai Hospital, Yilan 26546, Taiwan

**Keywords:** high-fructose diet, aquatic exercise training, aerobic exercise, body fat

## Abstract

Gradual weight gain in modern people and a lowering onset age of metabolic disease are highly correlated with the intake of sugary drinks and sweets. Long-term excessive fructose consumption can lead to hyperglycemia, hyperlipidemia and accumulation of visceral fat. Abdominal obesity is more severe in females than in males. In this study, we used a high-fructose-diet-induced model of obesity in female mice. We investigated the effects of aquatic exercise training on body weight and body composition. After 1 week of acclimatization, female ICR mice were randomly divided into two groups: a normal group (n=8) fed standard diet (control), and a high-fructose diet (HFD) group (n=24) fed a HFD. After 4 weeks of induction followed by 4 weeks of aquatic exercise training, the 24 obese mice were divided into 3 groups (n=8 per group): HFD with sedentary control (HFD), HFD with aquatic strength exercise training (HFD+SE), and HFD with aquatic aerobic exercise training (HFD+AE). We conducted serum biochemical profile analysis, weighed the white adipose tissue, and performed organ histopathology. After 4 weeks of induction and 4 weeks of aquatic exercise training, there was no significant difference in body weight among the HFD, HFD+SE and HFD+AE groups. Serum triglyceride (TG), AST, ALT, and uric acid level were significantly lower in the HFD+SE and HFD+AE groups than in the HFD group. The weight of the perirenal fat pad was significantly lower in the HFD+AE group than in the HFD group. Hepatic TG and total cholesterol (TC) were significantly lower in the HFD+AE group than in the other groups. Long-term intake of a high-fructose diet can lead to obesity and increase the risk of metabolic disease. Based on our findings, we speculate that aquatic exercise training can effectively promote health and fitness. However, aquatic aerobic exercise training appears to have greater benefits than aquatic strength exercise training.

## Introduction

Since modern people often eat out or eat too much, and the food is high in sugar and calories, many people gain weight day by day, and the age of onset of some diseases is falling. According to statistics from the World Health Organization (WHO), in 2008, it was estimated that 50 billion people were obese, and that number is increasing 1. The above phenomenon is associated with the excessive intake of sugar-sweetened beverages and sweets. It has been found in epidemiological research that the consumption of fructose in the diet, either sucrose or high-fructose syrup, is associated with metabolic-related complications, including obesity 2, cardiovascular disease 3, Type II diabetes mellitus 4, and non-alcohol fatty liver disease 5. Among the factors causing obesity, the primary ones are improper dietary behaviors and a lack of physical activity. Most people believe that obesity and metabolic diseases due to diet are caused by excessive fat intake, but they often ignore the fact that the calories from refined sugar are also actually one of the major causes of obesity. There is now strong evidence that intake of added fructose, contributes to the rising obesity and diabetes rates 6. Fructose is different from glucose in metabolism. Whereas glucose can be directly used as an energy source by human cells, fructose must be transported to the liver, kidney and intestinal tract and converted into glucose, lactic acids and fatty acids before being utilized by body tissues 7. Fructose may bypass the important rate-limiting step of glycolysis and enter the intermediate step of glycolysis in the metabolic process, leading to the production of excessive fatty acids 8. This production in turn leads to the accumulation of free fatty acids in the liver 9, 10. Furthermore, fructose is also insensitive to the stimulations of energy regulation hormones (including growth hormones, insulin and leptin), decreases metabolism and energy consumption, and increases appetite 11, 12. These special metabolic pathways easily cause an energy imbalance in the body and thus lead to metabolic disorders.

It has been found in both human clinical trials 11, 13 and animal experiments 14, 15 that excessive fructose intake is associated with different pathological-related symptoms, including weight gain, increased visceral fat, glucose intolerance, insulin resistance, hypertriglyceridemia and dyslipidemia. Many studies have confirmed the numerous health benefits of aquatic aerobic exercise training, including reducing visceral fat, increasing insulin sensitivity, and alleviating obesity and metabolic diseases caused by obesity. It is a very effective way to lose weight by improving the balance of hormones and metabolism in the body 16. However, strength exercise training in exercise therapy for weight loss in patients with metabolic diseases and obesity is also part of the exercise prescription. Fat oxidation can be promoted and lipid metabolism can be enhanced by aquatic strength exercise training, thereby reducing body fat and increasing muscle mass 17. It can effectively increase body mass, prevent diseases, and improve the quality of life 18. In this study, female mice were used to simulate human females consuming a high-fructose diet, and obesity was thus induced, to investigate whether body weight and body fat can be controlled by aerobic or aquatic strength exercise training and the effects of such training on dyslipidemia.

## Methods

### Animals and treatment

Female ICR mice (8 weeks old) grown under specific pathogen-free conditions were purchased from BioLASCO (Yi-Lan, Taiwan). All mice were provided a standard laboratory diet (No. 5001; PMI Nutrition International, Brentwood, MO, USA) and distilled water *ad libitum* and were housed under a 12-hr light/dark cycle at room temperature (22 °C ± 1 °C) and 50%-60% humidity. The Institutional Animal Care and Use Committee (IACUC) of National Taiwan Sport University (NTSU) inspected all animal experiments, and this study conformed to the guidelines of protocol IACUC-10527 approved by the IACUC ethics committee.

**Figure 1:**
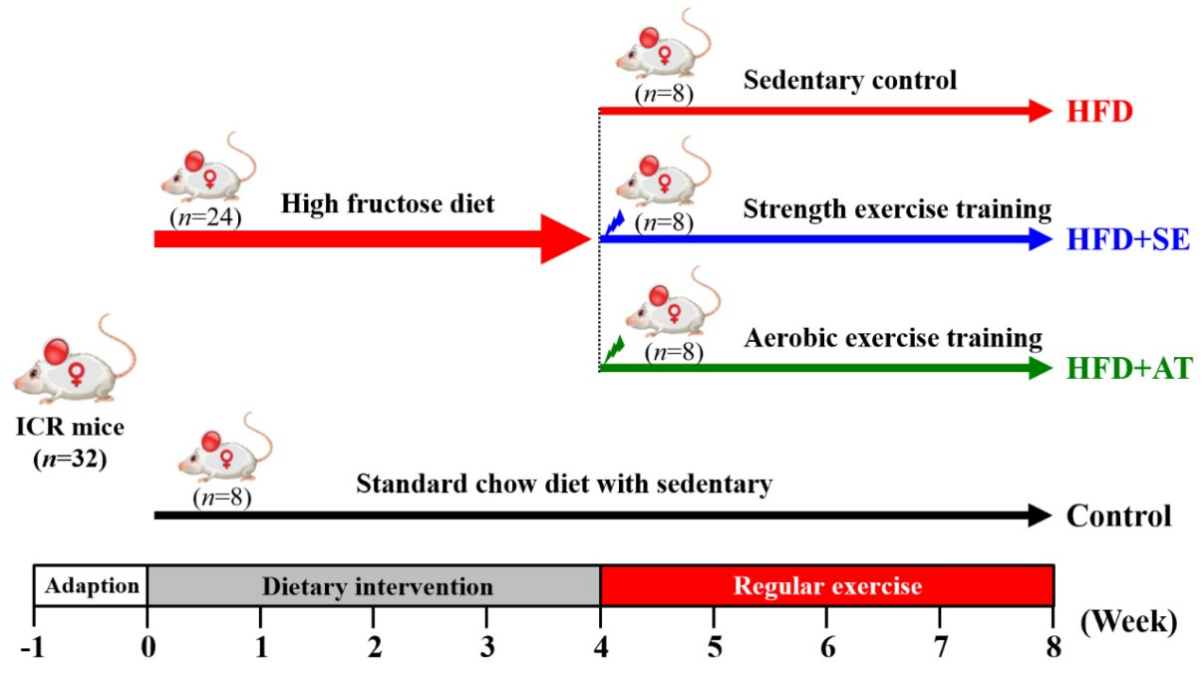
Experimental design.

The experimental design is depicted in Fig. 1. After 1 week of acclimatization, 32 mice were divided randomly into two groups. The normal group (n=8) was fed a standard chow diet (control), and the experimental group (n=24) received a high-fructose diet (HFD). The 24 experimental mice were divided into three groups (n=8/each group): (1) HFD with sedentary control (HFD), (2) HFD with aquatic strength exercise training (HFD+SE) and (3) HFD with aquatic aerobic exercise training (HFD+AE). Food intake and water consumption were recorded daily, and all animals were weighed weekly.

### Diet composition

The mice in the control group were fed a standard chow diet comprising 4.07 kcal/g. The calorie percentages were 28.5% protein, 13.5% fat and 58.0% carbohydrate. The mice in the high-fructose diet group were fed a high-fructose diet (HFD) comprising 4.22 kcal/g, 8% lard (wt/wt), 44% high fructose syrup (wt/wt), and 48% standard chow diet (wt/wt). The calorie percentages were 10.7% protein, 11.6% fat and 77.6% carbohydrate.

### Exercise training protocol

In the aquatic strength exercise training, progressive weight-bearing muscle strength training was used 19, with five training sessions per week. In the first week, the mice swam for 3 minutes and rested for 1 minute for six times in total, and the weight load was gradually increased from 3% to 8% of body weight. In the second week, the mice swam for 2 minutes and rested for 1 minute, and the weight load was increased by 3% of body weight every two days. For every increase of 6%, the number of times was doubled, and the swimming time decreased by 0.5 minutes. This pattern was repeated for the second to fourth weeks. An 86-liter bucket was used, the water temperature was 30 ± 1°C, and the water level was adjusted according to the length of each mouse. Eight experimental mice were put in a bucket, as described in a previous experiment 20.

In aquatic aerobic exercise training, the aquatic weight-free swimming training mode was used. The mice in the aquatic aerobic exercise training group received exercise training of 30 minutes per day, and the length was gradually increased by 3 minutes to 60 minutes per day. This swimming training mode without any weight was performed five times per week. According to the literature, the exercise intensity is between 40-60% VO2 max, so this mode is medium-intensity long-term exercise training 21.

### Clinical biochemical profiles

At the end of the experimental period, the mice were euthanized by 95% CO_2_ asphyxiation and blood was collected immediately. Serum was separated by centrifugation, and an autoanalyzer (Hitachi 7060) was used to measure the levels of the clinical biochemical variables: aspartate aminotransferase (AST), alanine aminotransferase (ALT), high-density lipoprotein cholesterol (HDL-C), low-density lipoprotein cholesterol (LDL-C), total protein (TP), blood urea nitrogen (BUN), creatinine, uric acid (UA), total cholesterol (TC), triacylglycerol (TG) and albumin.

### Hepatic lipid profiles

Hepatic lipids were extracted according to the method of Folch 22. In brief, extracted buffer A (chloroform : methanol = 2:1, v/v) was added to liver tissues, and the tissues were centrifuged at 3500 ×g and 4 °C for 10 minutes. After removal of the supernatants, the sample was remixed appropriately with extracted buffer B (chloroform : methanol : water = 3:48:47, v/v) and then centrifuged again. Finally, soluble lipids in the subnatant layer was used for further analysis. Hepatic triacylglycerol (TG) and total cholesterol (TC) concentrations were determined using diagnostic kits (Randox Laboratories, Antrim, UK). The liver extraction was mixed well with 100 times reagent buffer for 10 minutes and the absorbance value was measured under OD 500 nm.

### Histology of tissues

The liver, muscle (gastrocnemius and soleus muscles in the back part of the lower legs), heart, lung, kidney, uterine fat pads (UFP), retroperitoneal fat pad (RFP), and perirenal fat pad (PFP) from each group were photographed with a Cyber-Shot (DSC-HX30V, Sony, Tokyo). The tissues were dissected, weighed and snap-frozen in liquid nitrogen before being stored at -80°C. Another set of liver tissues was removed intact and fixed in 10% neutral buffered formalin for 24 hours before being processed for histopathologic analysis. Tissues were embedded in paraffin and cut into 4-μm thick slices for morphological and pathological evaluation, stained with hematoxylin and eosin (H&E), and finally examined by use of a light microscope equipped with a CCD camera (BX-51, Olympus, Tokyo).

### Statistical analysis

Data are expressed as mean ± SEM. Statistical differences were analyzed by one-way ANOVA with SAS 9.0 (SAS Inst., Cary, NC, USA). *P* < 0.05 was considered statistically significant.

## Results

### Effect of aquatic exercise training on body weight

**Figure 2:**
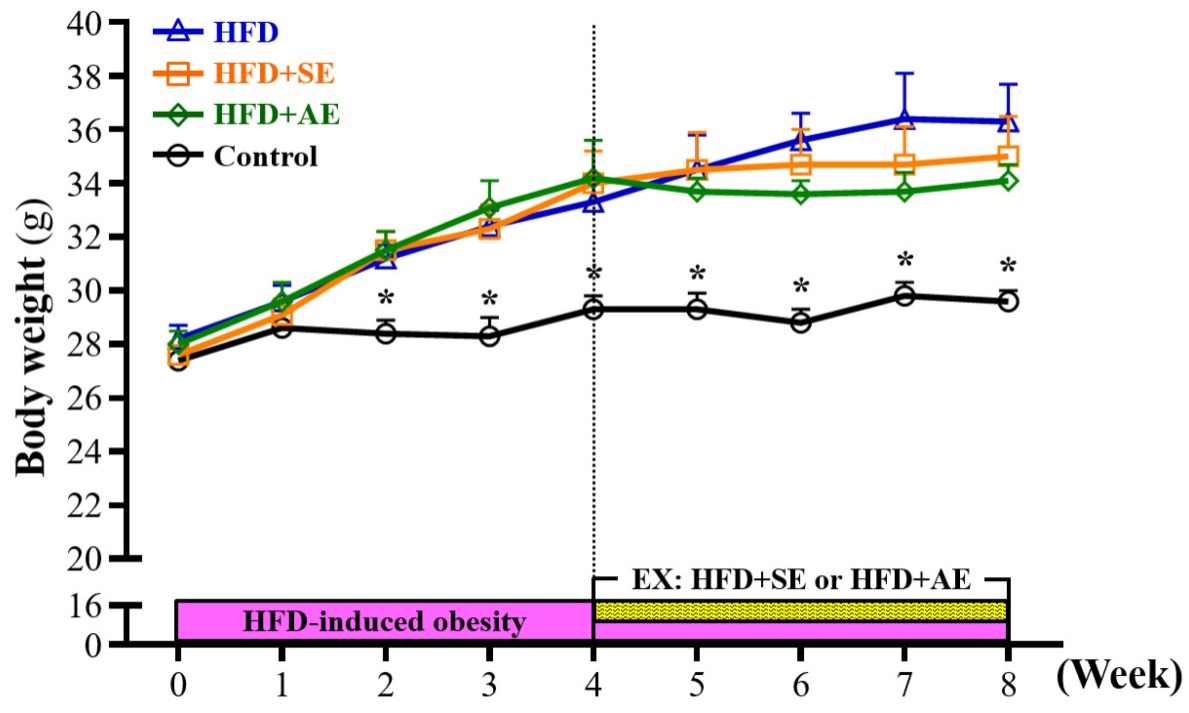
Aerobic or aquatic strength exercise training prevented high-fructose-diet-induced obesity in mice on a growth curve. Data shown are the mean ± SEM (n=8 mice per group). One-way ANOVA was used for analysis. * *P* < 0.05 compared with HFD group within each group.

The weekly weight changes of the animals in each group during the experiment are shown in Figure 2. Prior to the experiment, the mice were randomly divided into four groups: Control, HFD, HFD+SE, and HFD+AE. After the mice received a high-fructose diet, the body weights of the mice in the HFD, HFD+SE and HFD+AE groups were 10% (*P* = 0.0036), 11% (*P* = 0.0018) and 11% (*P* = 0.0017) higher than that in the control group respectively, and there were significant differences at the second week. The body weights of the mice in the HFD, HFD+SE and HFD+AE groups were 14% (*P* = 0.0015), 14% (*P* = 0.0016) and 17% (*P* = 0.0003) higher than that in the control group respectively, and there were significant differences at the third week. The body weights of the mice in the HFD, HFD+SE and HFD+AE groups were 14% (*P* = 0.0417), 16% (*P* = 0.0049) and 17% (*P* = 0.004) higher than that in the control group respectively, and there were significant differences at the fourth week.

### Effect of aquatic exercise training on water consumption and energy intake

**Figure 3:**
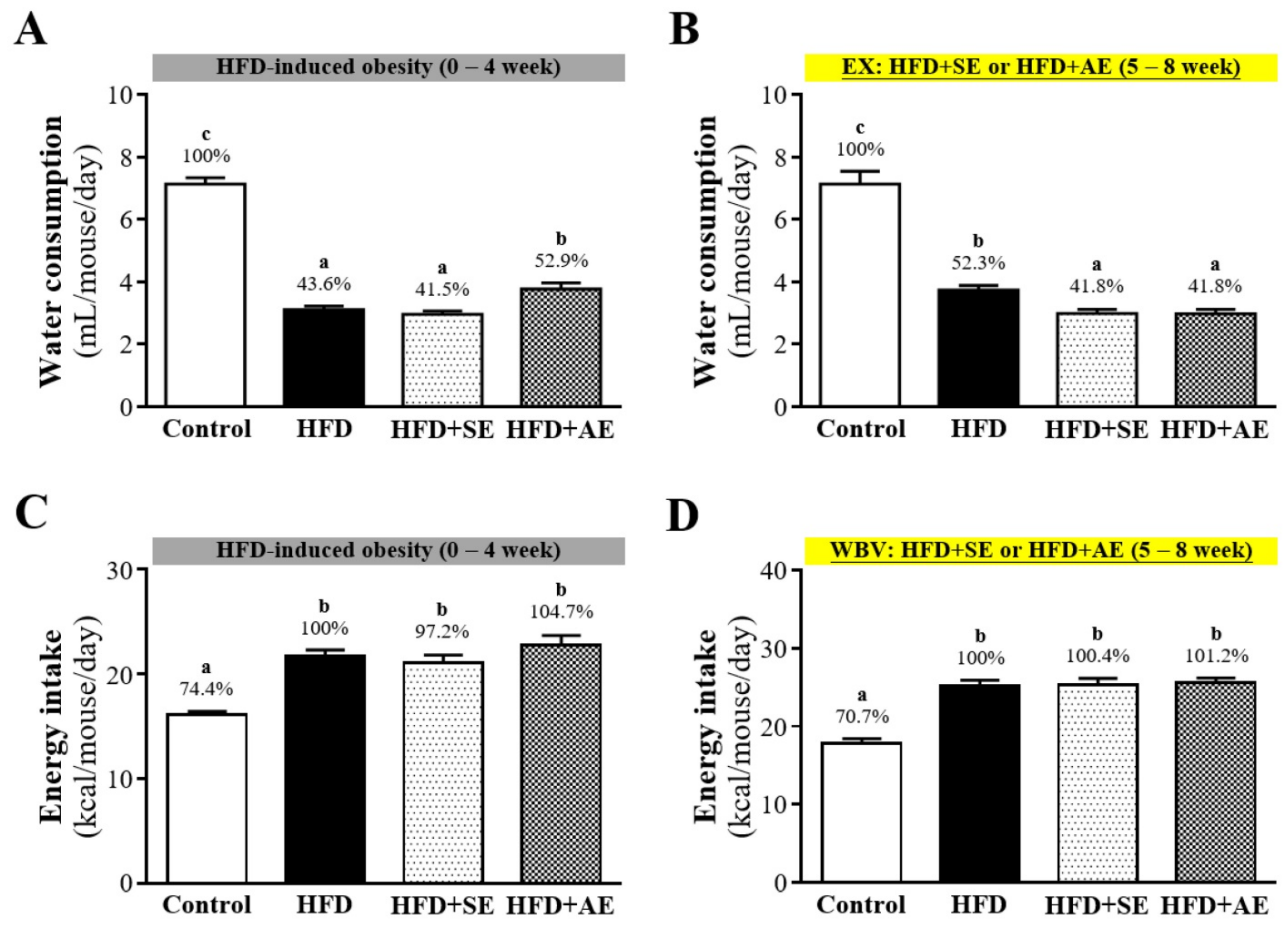
Aerobic or aquatic strength exercise training prevented high-fat-diet- induced obesity in mice on (A) water consumption and (B) energy intake. Mice (6 weeks old) were fed a HFD for up to 8 weeks combined with 4 weeks of aerobic or aquatic strength exercise training. Data shown are the mean ± SEM (n=8 mice per group). One-way ANOVA was used for analysis. * *P* < 0.05 compared with HFD group within each group.

The average daily food water consumption amounts in each group are shown in Figure 3A and 3B. In Figure 3A, during the first four weeks of high-fructose diet induction, the average daily water consumption amounts of each animal in the Control, HFD, HFD+SE and HFD+AE groups were 7.14 ± 0.20, 3.11 ± 0.12, 2.96 ± 0.11 and 3.77 ± 0.20 mL respectively, and the daily water intakes of the animals in the HFD, HFD+SE and HFD+AE groups were significantly lower than that in the Control group by 56.4% (*P* < 0.0001), 58.5% (*P* < 0.0001) and 47.1% (*P* < 0.0001). During the last four weeks of aerobic or aquatic strength exercise training, as shown in Figure 3B, the average daily water consumption amounts of each animal in the Control, HFD, HFD+SE and HFD+AE groups were 7.14 ± 0.41, 3.74 ± 0.15, 2.99 ± 0.13 and 2.98 ± 0.14 (mL) respectively, and the daily water intakes of the animals in the HFD, HFD+SE and HFD+AE groups were significantly lower than that in the Control group by 47.7% (*P* < 0.0001), 58.2% (*P* < 0.0001) and 58.2% (*P* < 0.0001).

The recorded data of the average daily calorie intakes of the animals in each group are shown in Figure 3C and 3D. In Figure 3C, during the first four weeks of the high-fructose diet, the average daily calorie intakes of each animal in the Control, HFD, HFD+SE and HFD+AE groups were 16.13 ± 0.30, 21.69 ± 0.61, 26.35 ± 0.9 and 28.39 ± 1.21 kcal respectively, and the daily calorie intake of the animals in the Control group was the lowest. As shown in Figure 3D, during second four weeks, with aquatic aerobic or aquatic strength exercise training, the average daily calorie intakes of each animal in the Control, HFD, HFD+SE and HFD+AE groups were 17.86 ± 0.54, 25.25 ± 0.67, 29.53 ± 0.92 and 21.50 ± 0.54 kcal respectively, and the daily calorie intake of the animals in the Control group was the lowest.

### Effect of aquatic exercise training on body fat

**Figure 4:**
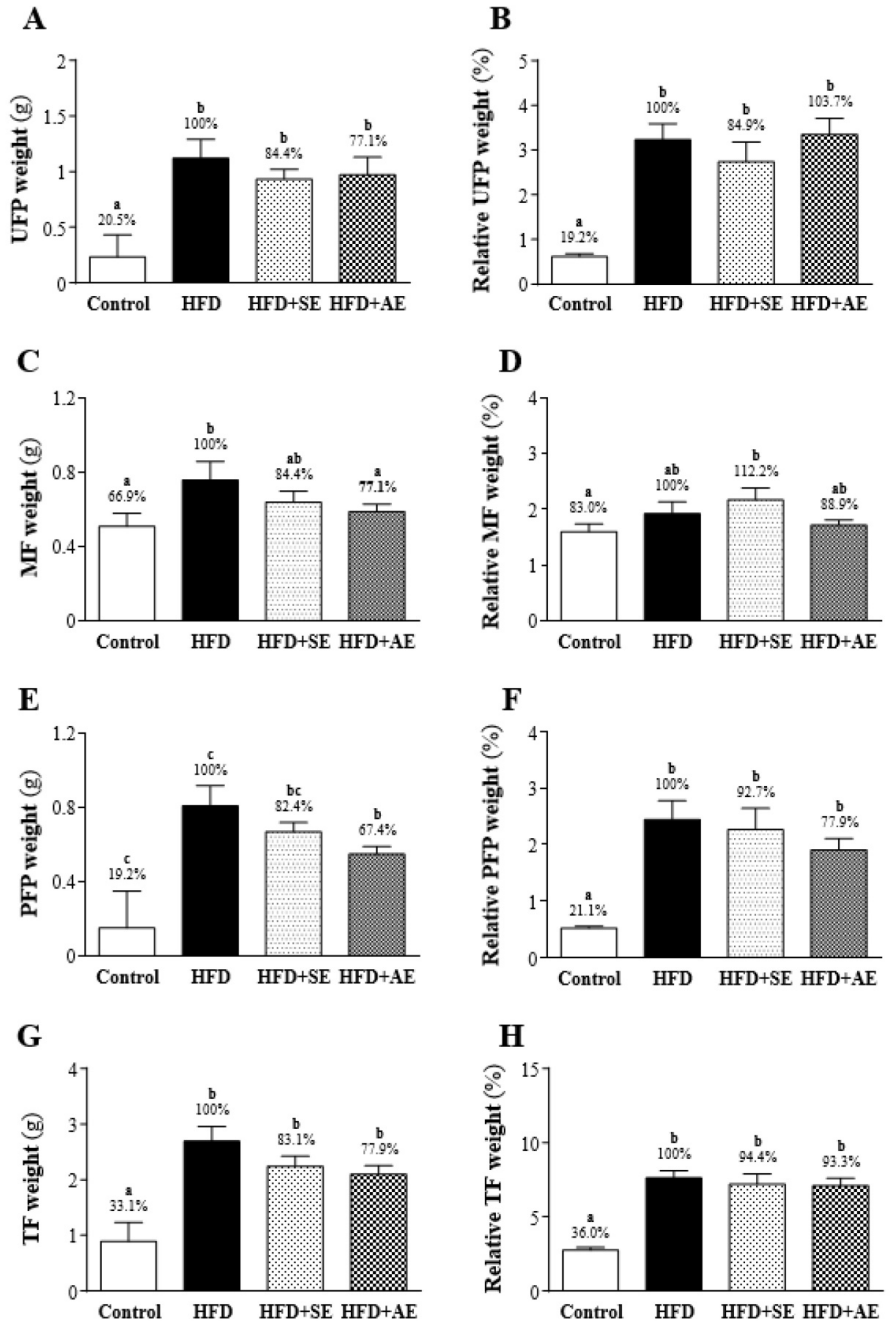
Effect of 4 weeks of aerobic or aquatic strength exercise training on tissue weights of (A) uterine fat pad (UFP), (B) mesenteric fat, (C) perirenal fat pad and (PFP) and (D) total body fat in HFD-fed mice. The relative weight (%) of (E) uterine fat pad (UFP), (F) mesenteric fat, (G) perirenal fat pad and (PFP) and (H) total body fat weight. Data are mean ± SEM (n=8 mice per group). Different letters (a, b, c) indicate significant difference at *P* < 0.05.

After the experimental mice were sacrificed, three sites were the primary collection sites of white adipose tissue: the uterine fat pad (UFP), the perirenal fat pad (PFP), and mesenteric fat (MF). As shown in Figure 4, the UFP, PFP and MF weights were significantly higher in the HFD group than in the Control group. As shown in Figures 4A, 4C and 4E, UFP, MF and PFP weights were higher in the HFD group than in the Control group by 79.5% (*P* < 0.0001), 33.1% (*P* = 0.0163) and 81% (*P* < 0.0001) respectively. In the HFD+SE and HFD groups, there were no significant differences in UFP, PFP and MF weights (*P* = 0.319, 0.2295, 0.2371). As shown in Figures 4C and 4E, comparing the HFD+AE and HFD groups, the weights of MF and PFP were significantly lower in the former group than in the latter group by 22.9% (*P* = 0.088) and 32.6% (*P* = 0.0309) respectively. There was no significant difference in the weight of UFP.

As shown in Figure 4G, the total body fat (TF; UFP+PFP+MF) weights were significantly higher in the HFD, HFD+SE, and HFD+AE groups than in the Control group by 66.9% (*P* < 0.0001), 50% (*P* = 0.0001) and 44.8% (*P* = 0.0005) respectively. There was no significant difference among the HFD+SE, HFD+AE and HFD groups.

As shown in Figures 4B, 4D and 4F, regarding the relative weights of each fat, UFP and PFP were significantly higher in the HFD group than in the Control group, but there was no significant difference in MF. The ratios of the relative weights of UFP, MF and PFP to the Control and the HFD groups were 80.8% (*P* < 0.0001), 17% (*P* = 0.1867) and 78.9% (*P* < 0.0001) respectively. There was no significant difference in UFP, PFP or MF (*P* = 0.3145, 0.6466, 0.3347) between the HFD and HFD+SE groups. Comparing the HFD group with the HFD+AE group, there was no significant difference in UFP, PFP or MF (*P* = 0.8013, 0.1705, 0.3791).

As shown in Figure 4H, the relative TF weights were significantly higher in the HFD, HFD+SE and HFD+AE groups than in the Control group by 64% (*P* < 0.0001), 58.4% (P <0.0001), and 56.3% (P < 0.0001) respectively. However, there was no significant difference between the HFD+SE, HFD+AE, and the HFD groups.

### Effect of aquatic exercise training on biochemical assessments

The contents of the blood biochemical parameters are shown in Table 1. AST and ALT were significantly higher in the HFD group than in the control group by 2 folds (*P* < 0.001) and 2.71 folds (*P* = 0.0018). AST and ALT were significantly lower in the HFD+SE and HFD+AE groups than in the HFD group.

The blood TG content was significantly higher in the HFD group than in the Control group by 1.54 folds (*P* < 0.001), but the blood TG contents in the HFD+SE and HFD+AE groups were not significantly different from that in the Control group. In the high-fructose diet groups, blood TG contents were significantly lower in the HFD+SE and HFD+AE groups than in the HFD group (*P* < 0.05). The blood TC contents were significantly higher in the HFD, HFD+SE and HFD+AE groups than in the control group by 1.99 folds (*P* < 0.001), 1.89 folds (*P* < 0.001) and 1.86 folds (*P* < 0.001). There was no significant difference between the high-fructose diet groups. The blood HDL-C contents were significantly higher in the HFD, HFD+SE and HFD+AE groups than in the control group by 2 folds, 1.74 folds, and 1.74 folds (*P* < 0.001). The blood LDL-C contents were significantly higher in the HFD, HFD+SE and HFD+AE groups than in the control group by 2.41 folds, 2.1 folds and 2.08 folds (*P* < 0.001), and there was no significant difference between the high-fructose diet groups.

The blood urea nitrogen (BUN) contents were lower in the HFD, HFD+SE and HFD+AE groups than in the control group by 59% (*P* < 0.001), 60% (*P* < 0.001), and 60% (*P* < 0.001). The creatinine (CREA) contents were lower in the HFD, HFD+SE and HFD+AE groups than in the control group by 83% (*P* = 0.0483), 78% (*P* = 0.0271), and 78% (*P* < 0.0092). The blood total protein (TP) contents did not differ significantly among the four groups. The blood albumin contents were significantly lower in the HFD group than in the control group by 94% (*P* = 0.0009). The blood uric acid (UA) contents were respectively lower in the HFD+SE and HFD+AE groups than in the control and HFD groups by 65% (*P* = 0.006) and 63% (*P* = 0.0031) and by 63% (*P* = 0.0072) and 64% (*P* = 0.0038).

### Effect of aquatic exercise training on liver TG and TC

**Figure 5:**
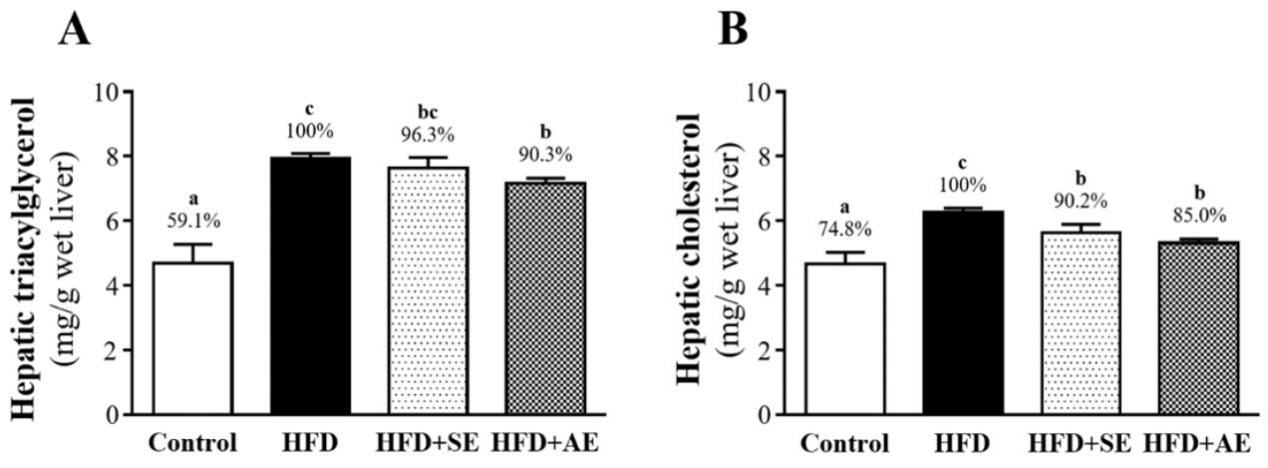
Aerobic or aquatic strength exercise training for mice: (A) liver, (B) skeletal muscle, (C) heart, (D) lung, (E) UFP, (F) kidney. In the fasted state, all the animals were sacrificed and tissue was removed for pathological analysis of tissue sections. Specimens were photographed by light microscopy. (Olympus BX51) (H&E staining, magnification: × 200, Scale bar, 40 μm).

As shown in Figure 5A, hepatic TG was significantly higher in the HED group than in the control (40.9%, *P* < 0.0001) and HFD+AE (9.7%, *P* = 0.0304) groups, and there was no significant difference between the HED and HFD+SE groups. Hepatic TC was significantly higher in the HFD group than in the control, HFD+SE and HFD+AE groups, by 25.2% (*P* < 0.0001), 9.8% (*P* = 0.0218) and 15.0% (*P* = 0.009), respectively (Figure 5B).

### Effect of aquatic exercise training on histology

**Figure 6:**
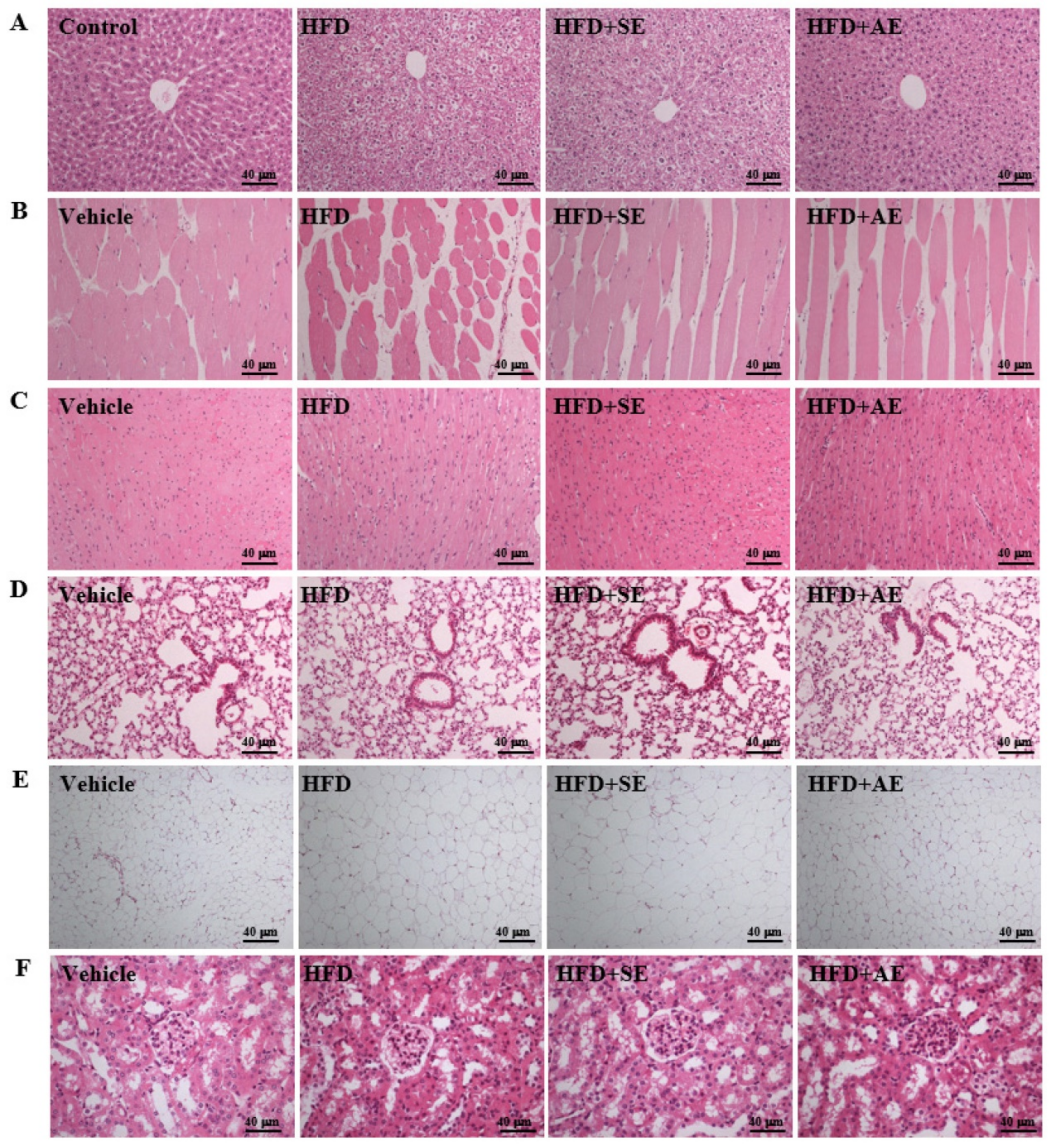
Aerobic or aquatic strength exercise training for mice: (A) liver, (B) skeletal muscle, (C) heart, (D) lung, (E) UFP, (F) kidney. In the fasted state, all the animals were sacrificed and tissue was removed for pathological analysis of tissue sections. Specimens were photographed by light microscopy (Olympus BX51) (H&E staining, magnification: × 200, Scale bar, 40 μm).

Fig. 6A presents H&E staining of the liver showing the sizes of the liver tissue cells in each group. Normal liver lobes have correct cell arrangement and no oil droplet accumulation (i.e., microvesicular fat). However, as compared to the Control group, the distribution proportion and size of lipid droplets in the HFD group were significantly greater, and as compared to the high fructose group, the distribution proportions and sizes of lipid droplets in the HFD+SE and HFD+AE groups were significantly smaller. Therefore, a decrease in the accumulation of microvesicular droplets and thus fatty liver resulted from the training of both exercise modes.

Figure 6B presents H&E staining of the gastrocnemius muscle showing that the cell size, muscle texture and arrangement of the gastrocnemius muscle were not abnormal in each group. Therefore, this experimental mode had no effect on the muscle tissues.

Figure 6C presents H&E staining of the heart showing that the cell sizes and arrangements of the cardiomyocytes were not abnormal in each group. Therefore, this experimental mode had no effect on the heart tissues.

Figure 6D presents H&E staining of the lung showing that the cell sizes and arrangements of the lung cells were not abnormal in each group. Therefore, this experimental mode had no effect on the lung tissues.

Figure 6E presents H&E staining of the UFP. In normal uterine tissues such as the Control group, the fat cells are tightly arranged and there is no vacuolar hypertrophy. However, as compared to those of the Control group, the size and proportion of adipose cells in the HFD group were significantly more hypertrophic. The vacuole proportion and cell size were smaller in the HFD+AE group than in the high fructose group, and there was no significant difference between the HFD and HFD+SE groups. These results indicate that aquatic aerobic exercise training can enhance the beta-oxidation of adipose tissues, thereby decreasing the fat proportion.

Figure 6F presents H&E staining of the kidney showing the renal cells. The arrangements and sizes of the renal glomerulus were not abnormal in the four groups. Therefore, this experimental mode had no effect on the kidney and functional diseases.

## Discussion

The results of this experiment showed that in ICR mice fed a high-fructose diet, the body weight significantly increased. The results confirm that consuming a high-fructose diet for four weeks can indeed cause obesity in mice. In the second week, the body weights of the three fructose diet groups (HFD, HFD+SE, HFD+AE) had all significantly increased as compared to the control group. After the four weeks of aquatic exercise training, the body weights of the HFD+SE and HFD+AE groups were 4.48% and 6.23% lower than that of the HFD group, respectively. There was a downward trend but no significant difference. When the mice received a high-fructose diet, their daily calorie intake was significantly higher than that in the control group. Studies have indicated that the intake of fructose can promote appetite and enhance dietary behaviors 23. The intake of fructose can inhibit growth hormones and reduce the concentration of leptin. The imbalance of these hormones can affect the regulation of appetite and lead to insufficient satiety, thereby increasing food intake 24. In the high-fructose diet groups, the daily calorie intake of the four-week aquatic aerobic exercise training (HFD+AE group) was significantly lower than that of the HFD group. It has been found by animal researchers that fructose intake is associated with leptin resistance and the occurrence of obesity 25, 26. A study by Astrup et al. found that after ten weeks of receiving a diet with 28% sucrose or artificial sweetener having equivalent calories, the group fed sucrose showed a significant increase in daily calorie intake, body weight and fat content 27. In terms of daily water consumption, it was obvious that the water intake of the ICR mice fed a high-fructose diet was lower than that of the control group, whether they were involved in aquatic exercise or not. It is presumed that due to the increase in daily food intake, the experimental mice were fully satiated, and thus the daily water intake decreased.

In terms of the adipose tissue content, after the eight weeks of fructose diet, the UFP, MF, PFP and TF weights were all significantly higher in the HFD group than in the control group, confirming once again that a high-fructose diet can indeed increase the occurrence of obesity. In terms of blood analysis, in the experimental group fed a high-fructose diet, the contents of TC, TG, LDL-C and HDL-C in the blood significantly increased. The literature has indicated that a large intake of fructose (including calorie-free beverages) can increase the risks of obesity 3, abdominal obesity 28, visceral fat 29, and dyslipidemia 30. Fructose can effectively induce the formation of endogenous fat in the liver and may increase the concentrations of triglyceride-rich lipoprotein (TRL-TG) and blood triglycerides after a meal 31. Previous studies have indicated that aquatic aerobic exercise training has an effect on reducing triglycerides, and that the visceral and abdominal adipose tissues of overweight and obese people also can be reduced 32, 33. The same results were found in this study. After aquatic aerobic exercise training, PFP was significantly lower in the HFD+AE group than in the HFD group, and the blood triglyceride content was also lower. The results of UFP tissue sections in the HFD+AE group showed that the proportions of vacuoles and cell sizes were smaller than those in the HFD group. A study by Egli et al. reported that, in a comparison of healthy subjects fed a high-fructose diet and doing 30-minute aquatic aerobic exercise training per day and those fed a high-fructose diet but no exercise, the concentrations of TG, TRL-TG and apoB48 in the blood were lower in the former, and the lipid oxidation and concentration of non-esterified fatty acids in the blood increased in the exercise group 34. Matos et al. demonstrated that aerobic exercise reduces the pro-inflammatory proteins levels and protein-tyrosine phosphatase 1B (PTP-1B) activity, thereby reducing the insulin resistance state 35, 36. Therefore, aerobic exercise increases insulin sensitivity and glucose uptake in skeletal muscle 37. On the other hand, exercise has been demonstrated to provide an agonist action of insulin in the skeletal muscle, extends to other key tissues such as the liver and adipose tissue 38, 39.

The results of this study show that a high-fructose diet can significantly increase the contents of liver damage indices, including AST and ALT, and the liver tissue sections showed that the distribution and size of lipid droplets in the livers of the HFD group were both significantly greater than those in the control group. These results indicate that, after the eight weeks of consuming a high-fructose diet, fat accumulation in the liver (i.e., fatty liver) developed in the mice, leading to liver dysfunction. Studies have indicated that fructose is the primary cause of excessive fat accumulation in the liver 40, 41. A high sugar diet (i.e., sucrose or high fructose corn syrup) can increase the risk of not only NAFLD 5 but also NASH 42.

The uric acid content in the blood was significantly reduced in the HFD+SE and HFD+AE groups. A previous study indicated that exercise is also one of the important factors that affect uric acid 43. Although doing vigorous aquatic aerobic exercise training for half an hour to three hours will increase the current uric acid content in the blood, a natural consequence of physical fatigue resulting from excessive exercise, long-term regular exercise can enhance physical fitness and reduce the uric acid content in the blood 44. Relevant studies have indicated that people with a higher frequency of activity have a lower incidence of hyperuricemia than do those with lower levels of activity or sedentary lifestyles 45. In a meta-analysis study of the correlation between physical activity and hyperuricemia, low-intensity and moderate-intensity physical activity were found to reduce the uric acid content by 12%, and high-intensity physical activity was found to reduce the uric acid content by 29% 46.

The long-term intake of a high fructose diet can cause obesity and increase the risk of metabolic diseases. In addition, the results of this study indicate that a long-term, continuous, non-improved diet cannot easily reduce the TC and LDL-C contents in the blood, even if exercise habits are increased. However, it seems that aerobic or aquatic strength exercise training has the potential to lower the risk of metabolic diseases. This study has found that body weight, as well as the TG, AST, ALT and uric acid contents of the PFP and in the blood, can be effectively reduced by aquatic exercise training. Further studies are needed to explore maintain of this program and follow up the period to validate the long-term of its benefits for health.

## Conclusions

After eight weeks of a high-fructose diet, the body weight, lipid content in the blood, and white adipose tissue content in the bodies (i.e., UFP, PFP, and MF) of the female mice were increased, as well as the distribution proportions and sizes of lipid droplets in the liver tissue and hypertrophy of adipose cells in the UFP tissue. However, there was a downward trend in weight, and the TG, AST, ALT and uric acid contents in the blood were reduced by the four weeks of aquatic strength and aerobic exercise training, as were the distributions and sizes of lipid droplets in the liver. The fat accumulation in the liver was also reduced, thereby decreasing the incidence of fatty liver. Furthermore, the sizes and vacuole proportions of the adipose tissues of the UFP can be significantly decreased by aquatic aerobic exercise training (HFD+AE group). Therefore, it seems that doing aquatic aerobic exercise training is more likely to reduce the occurrence of metabolic diseases than is doing aquatic strength exercise training. Additional studies on aquatic exercise training interventions for obesity women are recommended in the future.

## Figures and Tables

**Table 1:** Biochemical analysis at the end of the experiment.

Parameters	Control	HFD	HFD+SE	HFD+AE
AST (U/dL)	117 ± 14^ a^	235 ± 29 ^b^	143 ± 13^ a^	147 ± 9^ a^
ALT (U/dL)	47 ± 5^ a^	128 ± 32^ b^	51 ± 5^ a^	52 ± 5^ a^
HDL-C (mg/dL)	43 ± 3^ a^	86 ± 4^ c^	75 ± 3^ b^	75 ± 4^ b^
LDL-C (mg/dL)	4.9 ± 0.3^ a^	11.8 ± 1.3 ^b^	10.3 ± 1.0^ b^	10.2 ± 0.5^ b^
TC (mg/dL)	80 ± 4 ^a^	159 ± 7 ^b^	151 ± 4 ^b^	149 ± 8^ b^
TG (mg/dL)	93 ± 3^ a^	143 ± 14^ b^	99 ± 5^ a^	97 ± 6^ a^
TP (g/dL)	5.0 ± 0.1^ a^	4.9 ± 0.1^ a^	5.1 ± 0.0^ b^	5.2 ± 0.1^ b^
BUN (mg/dL)	23.4 ± 1.37^ b^	13.7 ± 0.5^ a^	14.1 ± 0.7^ a^	14.0 ± 0.8^ a^
Creatinine (mg/dL)	0.23 ± 0.02^ b^	0.19 ± 0.01^ a^	0.18 ± 0.01^ a^	0.18 ± 0.01^ a^
UA (mg/dL)	2.86 ± 0.41^ b^	2.95 ± 1.07^ b^	1.86 ± 0.13^ a^	1.89 ± 0.21^ a^
Albumin (g/dL)	3.44 ± 0.06^ a^	3.24 ± 0.03^ b^	3.43 ± 0.03 ^a^	3.49 ± 0.03^ a^

Data shown are the mean ± EM (n=8 mice per group). One-way ANOVA and Duncan's test were used for analysis. Different letters indicate significant difference at *P* < 0.05. AST, aspartate aminotransferase; ALT, alanine aminotransferase; TP, total protein; BUN, blood urea nitrogen; UA, uric acid; TC, total cholesterol; TG, triacylglycerol.
